# Global exposure to flood risk and poverty

**DOI:** 10.1038/s41467-022-30725-6

**Published:** 2022-06-28

**Authors:** Thomas K. J. McDermott

**Affiliations:** grid.6142.1 0000 0004 0488 0789 J.E. Cairnes School of Business and Economics, National University of Ireland Galway, Galway, Ireland

**Keywords:** Environmental economics, Natural hazards, Climate-change adaptation, Developing world

## Abstract

Flooding is a pervasive natural hazard, with new research demonstrating that more than one in five people around the world live in areas directly exposed to 1-in-100 year flood risk. Exposure to such flood risk is particularly concentrated amongst lower income households worldwide.

## Flooding as a pervasive risk

Among climate hazards, flooding is by far the most pervasive risk globally^1,2^. Tens of millions of people around the world are displaced from their homes by flooding each year, while damages from flood events run into the hundreds of billions of U.S. dollars in direct asset losses annually^3,4^. The human impacts of flooding tend to be concentrated disproportionately among low-income households, with vulnerability linked to poverty^5^. Alongside immediate impacts on people and their assets, floods may impose longer-term effects on welfare, constraining development opportunities of affected communities, particularly where coping capacity is limited and risks are not fully insured^6,7^.

Flood risk is expected to worsen in the coming decades, as rainfall intensifies and sea levels rise due to climate change^8^. A perhaps less well-recognized driver of increasingly costly flooding is the increase in exposure over time. Economic and population growth means there are more people and assets in harm’s way. Global development trends are also such that the fastest rates of economic development and population growth are often in places such as large coastal cities, where flood risks are particularly concentrated and getting progressively worse. This all means that the global economic costs and human impacts of flooding are likely to rise, perhaps substantially, over the coming decades. Development planning needs to account for these rising risks. This underscores the need to develop a better understanding of current exposure and vulnerability to climate hazards, as a prerequisite for informing sustainable and climate-resilient forms of development.

## New findings highlight the scale of the challenge

New research by Jun Rentschler of the World Bank, and colleagues, documents global exposure to flood risk at a very high spatial resolution. The authors combine state-of-the-art information on flood risk with high-resolution population maps to estimate exposure to flood risk on a global grid at 3 arc-seconds resolution (roughly 90 m × 90 m at the equator). The paper also adds subnational information on poverty rates and incomes, enabling Rentschler and colleagues to present the first global estimates of the interaction between exposure to flood risk, and poverty.

The data show that 1.81 billion people (23% of the world population) are directly exposed to 1-in-100-year floods. The vast majority (89%) of these people live in low- and middle-income countries. In terms of regional distribution, Rentschler et al.’s results show that flooding is a near-universal global phenomenon; of the more than 2000 subnational regions analysed, only nine have less than 1% of their population exposed to flood risks.

Rentschler and colleagues also estimate that some $9.8 trillion of economic activity globally, equivalent to 12% of the gross global product in 2020, is located in areas directly exposed to flooding. In contrast to population estimates, exposure of economic activity—at least in absolute terms—is found to be heavily concentrated in higher-income countries. However, as the authors point out, these exposure estimates do not account for flood protection measures. Typically when floods occur, a greater fraction of exposed economic activity is lost in lower-income settings.

In terms of the interaction of flood risk and poverty, Rentschler and colleagues estimate that over 780 million people living on under $5.50 per day face high flood risk. In other words, the data suggest that four out of every ten people exposed to flood risk globally live on low incomes.

This study represents an important contribution to our understanding of the scale and distribution of flood risk around the world, as well as the significant overlap between areas at risk of flooding and the concentration of poverty. Rentschler and colleagues have also contributed to the literature by providing comprehensive estimates of exposure to flood risk that account for all sources of flood risk—fluvial, pluvial, and coastal—with global coverage. Importantly, the state-of-the-art data employed here—comprising of 38 billion data points, covering 7.9 billion people in 189 countries—has resulted in substantial updates on previous estimates of flood exposure. Moreover, the evidence presented suggests that the number of people living with the dual challenges of flood risk and poverty is substantially higher than previously thought.

## Why does it matter to be exposed to flood risk?

Why does it matter to be exposed to flood risk? A hard-nosed economics response might be that it does not. At least in theory, if the costs of living with flood risk are balanced against the benefits of living in these riskier locations (for example, better access to job markets in urban areas) then flood risk may not always represent an important public policy concern. However, there are various reasons to suspect over-exposure to flood risk due to, for example, taxpayers bearing some of the costs leading to a form of moral hazard, or outdated, costly or otherwise missing information on flood risk.

Rentschler and colleagues’ findings also underline the additional concern that exposure to flood risk is unevenly distributed across income groups. But unequal exposure is only one part of the inequality of flood risk. The consequences of living with flood risk are radically different depending on household income; frequency of occurrence, losses relative to household income, and mortality rates are all dramatically higher in low-income settings^5,6^. Moreover, households with low incomes tend to have difficulty coping with risk, resulting in potentially longer-term consequences for development prospects^7,9^.

The estimates of people living in poverty exposed to flood risk quoted in this study may well represent a lower bound on the true figures. By applying subnational income data to headcount estimates of exposure, Rentschler et al.’s study implicitly assume that flood risk exposure is distributed evenly amongst richer and poorer households within regions. In reality, it’s likely that flood risk exposure varies systematically with incomes at a local level. For example, if housing markets price flood risk, even to a limited extent, then households will sort over flood risk by incomes^10^. Similarly, there may be institutional or political reasons for systematic local variation in exposure to flood risk by incomes; for example, if certain neighborhoods benefit more from investments in infrastructure that help to mitigate flood risk, including but not limited to flood defenses, basic sewerage and drainage systems^11,12^.

Further research is required to better understand how local context, including institutions and policies, affects the distribution and scale of flood exposure, how this exposure evolves over time, and how it might be expected to vary under different climate scenarios.

## Future prospects for flood mitigation

Rentschler and colleagues point to the need for investment in flood mitigation, including flood defenses, and identify hotspots where flood risk and poverty (or more generally vulnerability) coincide, and which might be considered priorities for investment.

However, flood defense systems are costly, take a long time to build, and come with additional challenges given uncertainty about the scale of future flood risk in the context of climate change^13,14^. Defensive systems will work better in some places than others, with some locations requiring more creative solutions such as green infrastructure or complementary investments in people, communities, and their capacity to cope with risk^7^. For some locations and threats, the optimal response may involve restricting the development of risky areas, allowing space for flood waters, and in some cases managed retreat.

Thinking about the threat of climate change more broadly, there is an expectation that as climate change alters risk profiles, people and economies will adapt to minimize the costs of these changes; including by relocating away from areas where risks are increasing^15^. For example, it has been shown that with free mobility, expected climate damages can be reduced dramatically^16^. However, various barriers and frictions constrain human (and economic) mobility; aside from political and legal restrictions on mobility, an equally significant constraint for many vulnerable households may be the financial cost of migration and associated risk^17,18^. It has been well documented that often the poorest and most vulnerable, or those directly affected by climate shocks, do not migrate^19,20^. The findings presented in Rentschler et al.’s research highlight that those most exposed to climate risk—in this case in the form of flood risk—are precisely those who may have the least capacity to adapt or move.
